# NF-κB Signaling in Tumor Pathways Focusing on Breast and Ovarian Cancer

**DOI:** 10.3389/or.2022.10568

**Published:** 2022-10-03

**Authors:** Monika Devanaboyina, Jasskiran Kaur, Emma Whiteley, Leslie Lin, Katelyn Einloth, Susan Morand, Laura Stanbery, Danae Hamouda, John Nemunaitis

**Affiliations:** ^1^ Department of Medicine, University of Toledo College of Medicine and Life Sciences, Toledo, OH, United States; ^2^ Gradalis, Inc., Carrollton, TX, United States

**Keywords:** ovarian cancer, breast cancer, NFκB, immunotherapy, tumor signaling, inflammatory signaling

## Abstract

Immune disorders and cancer share a common pathway involving NF-κb signaling. Through involvement with GM-CSF, NF-κB can contribute to proliferation and activation of T- and B- cells as well as immune cell migration to sites of inflammation. In breast cancer, this signaling pathway has been linked to resistance with endocrine and chemotherapies. Similarly, in ovarian cancer, NF-κB influences angiogenesis and inflammation pathways. Further, BRCA1 signaling common to both breast and ovarian cancer also has the capability to induce NF-κB activity. Immunotherapy involving NF-κB can also be implemented to combat chemoresistance. The complex signaling pathways of NF-κB can be harnessed for developing cancer therapeutics to promote immunotherapy for improving patient outcomes.

## Introduction

Nuclear factor kappa-light-chain-enhancer of activated B cells (NF-κB) comprises a group of transcription factors which play an important role in mediating inflammatory signaling pathways. As NF-κB signaling dysregulation is correlated with immune disorders and cancer, the pathway is vital to tumorigenesis, oncologic development and developing therapeutics [1, 2].

NF-κB signaling includes the canonical and non-canonical pathways. The canonical pathway is dependent on Nuclear factor kappa beta Essential Modulator (NEMO or IKKγ) activation and subsequent kinase complexes such as inhibitory kappa beta kinase alpha and beta (IKKα and IKKβ). NEMO’s ubiquitin domain can recruit IKK for degradation which decreases inhibition of NF-κB to participate in inflammatory signaling. Activation of the canonical pathway occurs through cytokines, specifically tumor necrosis factor (TNF) and IL (Interleukin)-1, pathogen-associated molecular patterns (PAMPs), and other immune signals. Canonical pathway activation leads to RelA and RelB transcription factor activation.

The non-canonical pathway is NEMO independent and plays a significant role in activating TNF receptors including CD40, LTßR, RANK, and TNFR2. NF-κB-inducing-kinase (NIK) is the major inducer in non-canonical signaling. This pathway is correlated with development of the lymphoid system through B cell survival and maturation along with dendritic cell activation [2].

In addition, NF-κB has shown the capability to affect cell survival and proliferation which could affect the tumor microenvironment and pathogenesis. Increased expression of NF-κB and associated signaling molecules is associated with poor prognosis in many cancer types, such as bladder and non-small cell lung cancer [3, 4]. Recent literature has shown that increased expression of NF-κB may contribute to ovarian cancer [1].

## NF-κB Role in GM-CSF Pathway

Granulocyte monocyte colony stimulating factor (GM-CSF) has the capability to activate NF-κB [5, 6]. This activation of NF-κB occurs through the canonical pathway which also induces the formation p52 and RelB heterodimers [7]. NF-κB is bound by the inhibitor of kappa B (IκB) complex in the inactive state. As IκB is phosphorylated, NF-κB is released and may translocate to the nucleus to induce transcription of immune and inflammatory cytokines [8]. The ɑ- and β-chains of GM-CSF receptor interact with IκB kinase beta (IκKβ), a component of IκB kinase complex, which normally inhibits NF-κB. With GM-CSF, the GM-CSF receptor-ɑ chain activates IκKβ which ubiquitinates and inactivates IκB, releasing NF-κB [7, 9]. Further analysis illustrated other possible mechanisms including TNF receptor-associated factor 6 (TRAF6) mediation in GM-CSF activation of NF-κB. TRAF6 directly activates IκK leading to the disinhibition of IκB and activation of NF-κB [7].

NF-κB can activate multiple downstream signaling pathways, including the proliferation, differentiation, and activation of T- and B-cells [10]. NF-κB regulates transcription of pro-inflammatory cytokines such as TNF-α, increasing the role of NF-κB in inflammation [11]. In this sense, GM-CSF’s role with NF-κB increases the functional activity of effector lymphocytes, while also increasing pro-inflammatory cytokines. NF-κB also interacts with vascular endothelial cells to improve immune cell migration *via* enhanced expression of adhesion molecules on leukocytes and endothelial cells, to allow circulating leukocytes to enter the site of inflammation [12]. GM-CSF has also been shown to stimulate anti-tumor immune responses through dendritic cell activation and T-lymphocyte activity [6]. This review article more comprehensively details the role of NF-κB signaling in breast and ovarian cancer to highlight possible upcoming therapeutic approaches.

## Role of NF-κB in Breast Cancer

Breast cancer is the fourth leading cause of cancer death in the United States and it is predicted that in 2019, there were approximately 268,600 cases of invasive breast cancer [13]. Breast cancer is a heterogeneous disease stratified by hormone receptor and human epidermal growth factor (ErbB2/(Her2/neu)) receptor status. Tumors negative for both hormone and epidermal growth factor receptors are called triple negative breast cancer (TNBC) and are common in women with a *BRCA1* mutation [14, 15].

**FIGURE 1:**
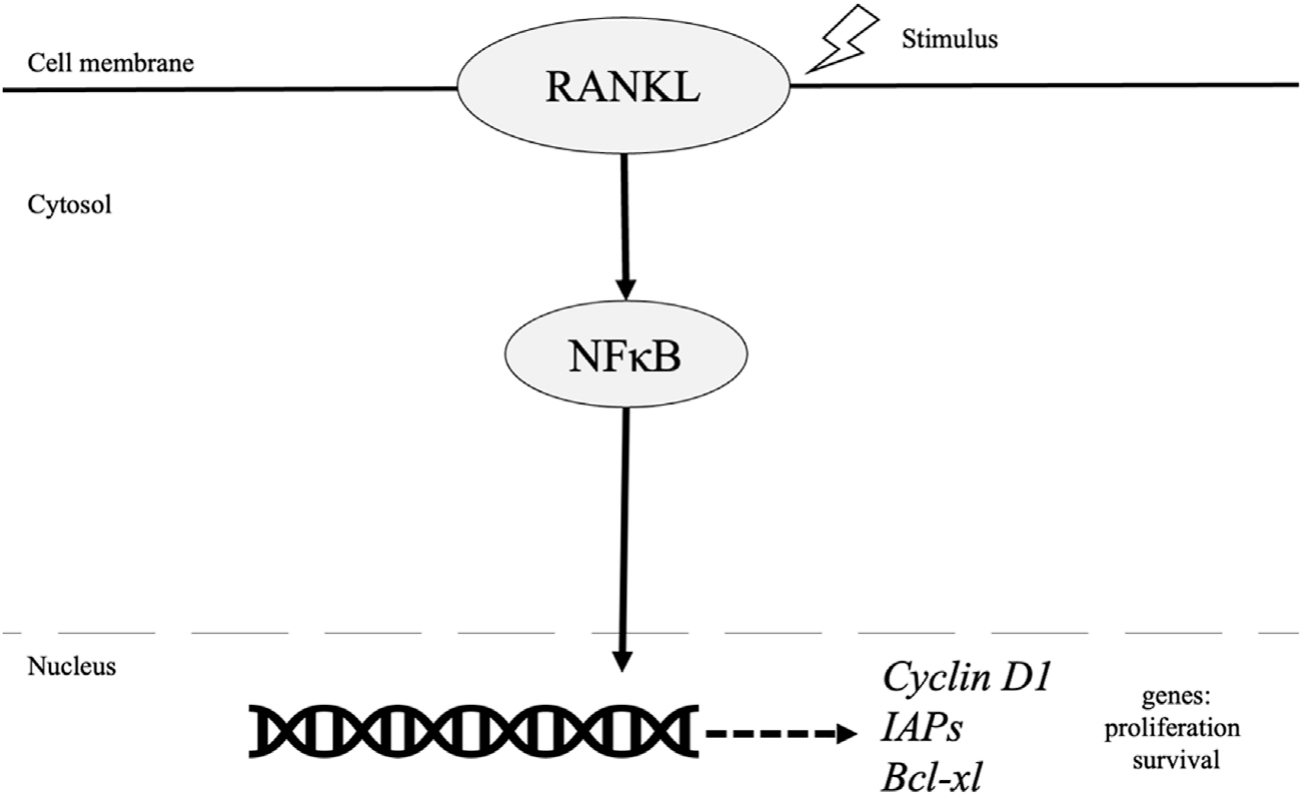
Role of NF-κB pathway in breast cancer.

While these tumor types are distinct, constitutive activation of NF-κB is a frequent characteristic found in most breast cancer tumors as well as other cancer types. In breast cancer specifically, NF-κB is present at higher levels in grade III tumors at 86.9% and only 37.5% in grade I tumors (*p* = 0.002) [16]. NF-κB plays an essential role in normal mammary gland development mediated by receptor activator of NF-κB ligand (RANKL), its receptor RANKL, and the decoy receptor osteoprotegerin (OPG). RANKL activates NF-κB, inducing cellular proliferation by targeting cyclin D1 as illustrated in Figure 1 [17] while also while also protecting cells from apoptosis, and assisting with tumor cell renewal (Suarez 2010, Wang 2015). In fact, A study by Kiechl et al, concluded serum levels of RANKL/OPG are potential predictors of predisposition and prognosis of breast cancer specially in post-menopausal women (Kiechl, 2017).

Survival is also mediated by NF-κB through increased inhibitors of apoptosis (IAPs) and Bcl-xL in breast cancer cells [18]. Inactivation of NF-KB *via* downregulation of Transglutaminase 2 (TGase2), a cross linking enzyme, leads to apoptosis of drug resistant cancer cells. Furthermore, TGase 2 gene silencing led to decreased expression of survival factors such as Bcl-xL and Bcl2 [19-21]. Additionally, previous evidence has illustrated NF-κB activation and its role in driving breast cancer development and progression *via* its role in cancer stem cells (CSC), epithelial to mesenchymal transition (EMT) and resistance to endocrine and chemotherapies.

Cancer stem cells (CSC) play an imperative role in tumor initiation and act to drive tumorigenesis and treatment resistance. CSCs express epithelial-specific antigen (ESA) and CD44. In one study, suppression of NF-κB led to dramatically decreased proportions of CD44-positive cells in Her2 dependent tumors. Her2, overexpressed in one-third of all breast cancers, has also been shown to control the CSC population. Specifically, Her2 can activate NF-κB through the canonical pathway. NF-κB was shown to influence tumor initiation, cell proliferation and recruitment of tumor associated macrophages (TAMs) in a HER2 mouse model after selective suppression of NF-κB [18].

Metastasis is largely driven in solid tumors by the epithelial-to-mesenchymal transition (EMT) [22]. Previous research has established the role of NF-κB in the induction and maintenance of EMT, an imperative process for breast cancer progression [23, 24]. Several mechanisms by which NF-κB influences EMT include NF-κB/p65 as a transcriptional regulator of EMT transcriptions factors such as SLUG, SIP1, and TWIST1 as well as through NF-κB dependent expression of ZEB-1/ZFHX1A and ZEB-2/ZFHX1B/Smad-interacting protein [25]. Dysregulation of the RANKL/RANK/OPG system and its relationship to metastatic bone disease has been previously documented (Infante, 2019). Treatments such as denosumab, a human monoclonal antibody, which inhibits RANKL, is currently used [26, 27]. RANKL/RANK system induced EMT has been linked to upregulation of Snail and TWIST1 and downregulation of E-cadherin [28]. In addition, TWIST1 was observed in bone marrow of breast cancer patients and its expression correlated with occurrence of metastasis [29].

Furthermore, inhibition of NF-κB/p65 with dehydroxymethylepoxyquinomicin (DHMEQ) decreased migration and invasion in human breast cancer cell lines, MDA-MB-231 and HCC-1954 [30]. Another study also validated these results as DHMEQ inhibited 3D invasion of breast carcinoma cells through inhibition of matrix metalloproteinase (MMP), a peptidase important for extracellular matrix degradation in the tumor microenvironment, along with inhibition of IL-6 [31].

Previous studies have associated NF-κB activation in estrogen receptor positive (ER+) tumors and is associated with resistance to endocrine and chemotherapies [32, 33]. Approximately 80% of breast cancer are ER+ and bind estrogen to stimulate cancer cell growth. Typically, drugs such as tamoxifen and aromatase inhibitors, which inhibit estrogen receptors or lower estrogen levels, respectively, are used to treat ER + breast cancer. However, disease recurrence and resistance to treatment is common [34]. A cohort study in Indonesia, examined NFKB expression, ER status and HER2 status as potential predictors of response to a chemotherapy regimen of cyclophosphamide-doxorubicin-5FU (CAF). Patients with negative NFKB expression were 10-times more likely to be responsive to chemotherapy compared to those with positive NFKB expression [35]. Potential mechanisms for resistance are discussed below. The various implications of NF-κB in the context of breast cancer make it an important therapeutic target.

## Role of NF-κB in Ovarian Cancer

Constitutive activation of NF-κB has also been linked to the development of epithelial ovarian cancer (EOC). Activation of the canonical NF-κB pathway can be triggered by an inflammatory stimulus after which cytokines such as TNFα, interleukin-1α/β bind to their receptors [36–38]. Binding of cytokines to their receptors leads to subsequent phosphorylation of NF-κB, which enables dimerization and translocation of RelA and p50 into the nucleus for initiation of downstream gene transcription involved with proliferation, invasion, adhesion, and angiogenesis [39-41]. Heterodimer p50 and c-Rel can also enter the nucleus to induce antiapoptotic gene transcription and regulate genes involved in cell cycle checkpoint inhibition [42, 43]. As a result, EOCs associated with NF-κB signaling tend to have a poor prognosis due to increased gene expression that are advantageous for malignant progression [44].

Interaction between NF-κB and estrogen has also been suggested to contribute to the pathogenesis of EOC. Estradiol regulates NF-κB by degrading inhibitor of nuclear factor κB (IkB) and preventing phosphorylation of IkB proteins by IkB kinase (IKK) complex [45]. The phosphorylation and subsequent degradation of IkB proteins ultimately leads to release of heterodimers capable of mediating gene transcription within the nucleus. Overexpression of estrogen receptor (ER) can also suppress NF-κB activity by blocking DNA-binding by NF-κB, inhibit gene transcription by binding to DNA-bound NF-κB, and decrease IL-6 production, another inflammatory mediator of NF-κB signaling [46, 47]. Despite this, constitutively active NF-κB signaling in gonadal cell tumors has been found to repress ER-mediated transactivation even in the presence of estradiol binding [48].

**FIGURE 2:**
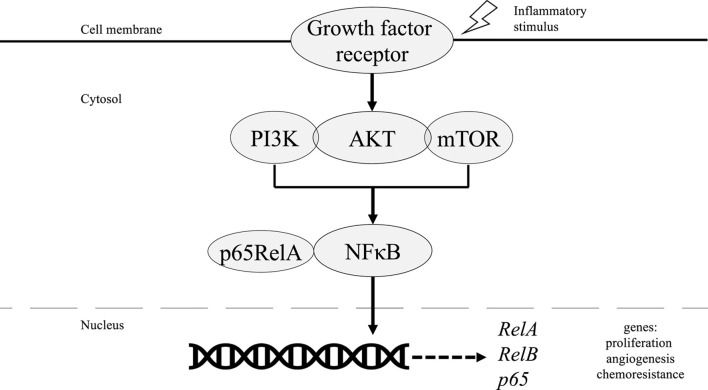
Role of NF-κB in PI3K/AKT/mTOR pathway in ovarian cancer tumorigenesis.

Most cases of ovarian cancer, however, occur further upstream *via* hyperactivation of the phosphoinositol 3 kinase/protein kinase B/mammalian target of rapamycin (PI3K/AKT/mTOR) pathway as shown in Figure 2. Overexpression of this pathway drives downstream activation of NF-κB which induces proliferation, angiogenesis, and expression of antiapoptotic genes responsible for the survival and aggressiveness of EOCs [49]. Triggered by the release of pro-inflammatory cytokines, activation of the PI3K/AKT/mTOR pathway is followed by phosphorylation of PI3K regulatory subunit p85, which subsequently increases the catalytic activity of PI3K-110α. PI3K-110α then phosphorylates downstream NF-κB subunit p65RelA, thus allowing NF-κB translocation into the nucleus [50, 51]. Moreover, NF-κB has binding sites in the promoter region of PIK3CA, an oncogene that encodes for PI3K-110α [52]. Therefore, reciprocal activation results in high expression levels of p65RelA that is associated with increased chemoresistance and poor outcomes in patients with EOC [53].

PI3K can also phosphorylate AKT which subsequently activates the IKK complex to phosphorylate p65RelA, which allows for nuclear translocation. For AKT to induce NF-κB activity *via* activation of IKK, however, requires assistance from downstream mTOR-associated protein Raptor which interacts with and stimulates IKK [54]. Otherwise, AKT can directly phosphorylate and activate the p65RelA subunit without IKK activity [55]. The downstream pathway of NF-κB increases the gene RelA and co-expression of RelB. This leads to the increase of the gene p65, enhancing spheroid growth in ovarian cancer cells [56]. Preclinical models have shown decreased RelB expression with doxycycline treatment in ovarian cancer cells in addition to significantly decreased spheroid formation, possibly due to increased c-myc-cyclins D1 and E [1, 56]. The absence of RelB also decreased ovarian cancer tumor metastasis to abdominal organs [56]. Another marker for tumorigenicity in ovarian cancer includes aldehyde dehydrogenase (ALDH). ALDH can be inhibited by silencing RelB, which indicates the significance of NF-κB activity in ovarian cancer. Decreasing ALDH with siRNA further decreased tumor formation and increased sensitization to treatment with carboplatin [56].

NF-κB can additionally mediate selective gene activation *via* regulation of Smad intermediaries of transforming growth factor-β (TGFβ) signaling. Smad proteins are a family of signal transducers that function as transcriptional regulators for TGFβ signaling, which is essential in the regulation of proliferation, differentiation, and apoptosis of cells. Co-expression and interaction between NF-κB subunits p52 or p65 with Smad3 or Smad4 not only enhance TGFβ transactivation activity, but also strongly increase basal transcription of target genes [57]. In turn, increased TGFβ activity can further induce NF-κB transcriptional activity. Smad7 activity, which normally inhibits TGFβ, is suppressed by p65 expression even without a consensus NF-κB binding site [58]. This results in dysregulation of TGFβ signaling that is essential to tissue maintenance and development and can ultimately lead to uncontrolled tumor growth and cell invasion.

Other mediators downstream of NF-κB include VEGF and IL-8 which play an important role in angiogenesis and tumor development[1]. In ovarian cancer, Cytokines IL-6 and IL-8 are activated by NF-κB andpromote tumor growth*via* immunosuppression. Inhibition of NF-κB leads to decreased levels of IL-6 and IL-8 in ovarian cancer cells indicating the direct correlation between NF-κB and ovarian cancer progression[59].

Along with downstream regulators, NF-κB influences upstream cellular signaling molecules. Upstream regulation of NF-κB is mediated through epidermal growth factor receptor (EGFR), a tyrosine kinase receptor that is increased in 70% of ovarian cancers. EGFR regulates cell growth on the epithelial surface of ovaries and development of ovarian follicles. Through NF-κB, EGFR upregulates proinflammatory markers, IL-6 and plasminogen activation inhibitor (PAI-1), leading to increased proliferation in ovarian cancer cells.Co-expression of IL-6 and PAI-1 in epithelial ovarian cells was significantly associated with advanced-stage EOC and decreased survival [60]. Silencing EGFR not only decreased NF-κB activity but also decreased IL-6 and PAI-1 activity[60].

Another upstream mediator in the NF-κB pathway involves tripartite motif (TRIM) genes with oncogenic capabilities. TRIM genes are responsible for encoding proteins in cell growth, development, and differentiation. Compared to normal tissue, there is increased TRIM52 expression in ovarian cancer cells after the phosphorylation of IKKB and p65 with NF-κB activation. Absence of TRIM52 resulted in decreased ovarian cancer growth and increased apoptosis through decreasing levels of NF-κB [61].

Understanding the mechanisms of the NF-κB pathway could lead to identification of key molecules or enzymes for targeted therapy in ovarian cancer. For example, anti-EGFR compounds and ALDH inhibitors in the NF-κB pathway have been utilized as therapeutic measures in ovarian cancer [56, 60]. Furthermore, polyphenols and curcumin have shown anti-cancer and anti-inflammatory effects through the regulation of NF-κB with miRNA expression [62, 63]. Weldolactone was found to also suppress NF-κB activity in ovarian cancer cell lines and decrease proliferative activity [64]. The development of targeted therapeutics combining NF-κB and anti-estrogen therapy should be further investigated to improve ovarian cancer outcomes.

## Chemotherapy Resistance in Breast and Ovarian

A major setback in the treatment of breast and ovarian cancers is resistance to chemotherapy. Resistance can occur intrinsically or can be acquired over time. The cell membrane plays an important role through absorption of chemotherapy and efflux mechanisms such as P-glycoprotein, along with enzymes inside the cell that can alter metabolism [65]. Previous studies have shown that breast cancer resistance could be affected by growth factor signaling mechanisms. Alterations in the PI3K/AKT/mTOR and RAS/MAPK/ERK signaling pathways have been related to resistance with endocrine therapy like tamoxifen [66]. NF-κB plays a key role in the PI3K pathway and similar effects could be noted with NF-κB.

Although chemoresistance may be multifactorial, activation of NF-κB is one mechanism linked to such resistance. Common chemotherapies, such as anthracyclines, including doxorubicin are shown to activate NF-κB and its pro-survival downstream targets which contribute to chemoresistance. The mechanisms by which doxorubicin activates NF-κB remain unclear, however previously studied mechanisms include activation *via* the IKK complex, PI3K dependent pathway, and c-Abl kinase activity in breast cancer cells [67, 68]. Microtubule disrupting chemotherapies, including taxanes, platinum agents, and vinca alkaloids have also been shown to activate NF-κB [18, 69].

A study using NF-κ B/p65 nuclear translocation staining as a measure of NF-κB activation found that nuclear immunohistochemical staining was significantly correlated with resistance to neoadjuvant chemotherapy in breast cancer patients [70]. Additionally, NF-κB activation was increased in patients after chemotherapy, suggesting that NF-κB is inducible by chemotherapy. Increased NF-κB activity, measured by NF-κ B/p65, was seen in ovarian cancer cell lines with resistance to the chemotherapy agents such as platinum, paclitaxel, and erlotinib [1].

## *BRCA* Signaling

Breast cancer susceptibility genes 1 or 2 mutations (*BRCA1/2*) are linked with hereditary breast and ovarian cancers (HBOC), 5%–10% of breast cancers and 10%–15% of ovarian cancers possess a *BRCA1/2* mutation [71]. The estimated lifetime risk of developing breast cancer is 40%–80% in patients carrying either *BRCA1/2* mutation. The estimated lifetime risk of developing ovarian cancer is 25%–65% and 15%–20% in patients with *BRCA1* and *BRCA2* mutations, respectively [72]. *BRCA1* is a multifunctional protein with important roles in cell-cycle control, ubiquitination, transcriptional regulation, DNA damage repair [73, 74].

Although less common, NF-κB activation can also occur by DNA damage. Mutations in the *BRCA1* tumor suppressor gene are often found in EOCs. *BRCA1*-null mutations in ovarian cancer cells induce NF-κB signaling, which causes enhanced transcriptional activation of target genes that promote increased autophagy, glycolysis, and oxidative stress in the stroma [75]. The increased levels of reactive oxygen species (ROS)can then further upregulate NF-κB [76]. When *BRCA1*-null ovarian cancer cells were rescued with the wild-type, *BRCA1* overexpression successfully blocked NF-κB activation and decreased oxidative stress within the microenvironment [75]. These findings demonstrate the important regulatory role BRCA1 plays in NF-κB activation. Therefore, mutation of *BRCA1* can lead to pathway dysregulation further downstream and repression of BRCA1-induced apoptosis, further making chemotherapy treatment of EOCs challenging [77].

BRCA1 was found to bind the p65/RelA subunit of NF-κB to stimulate tumor necrosis factor-alpha (TNF-α) and interleukin-1 (IL-1) [78]. A study by Buckley et al. discovered consistent NF-κB hyperactivity associated with *BRCA1* dysfunction as a consequence of increased reactive oxygen species (ROS). Higher NF-κB activity was found in *BRCA1*-mutant and *BRCA1*-low cells compared to their isogenic matched *BRCA1* reconstituted controls. ShRNA mediated *BRCA1* knockdown in a normal breast cell line also led to increased NF-κB activity. Further investigation into how *BRCA1* function leads to basal NF-κB hyperactivity was performed using a series of inhibitors to pathways known to be regulated by *BRCA1* activity. These pathways include: Notch, DNA Damage Response (ATM and PARP inhibitors), and ROS. Of interest, inhibition of ROS resulted in loss of increased NF-κB activity seen in *BRCA1* dysfunction. ROS levels were significantly higher in cells lacking functional *BRCA1*. To determine whether the same biology can be observed in breast cancer cells, a cell line-derived gene list of target genes upregulated by NF-κB with dysfunctional *BRCA1* was created. The list was refined to contain genes with the most significant fold changes. The gene list was used to interrogate TNBC microarray dataset enriched for *BRCA1* mutations to identify a subgroup of breast cancers labeled as *BRCA1*(-)/*NF*-*κB*(+) (“*NF-κB* on”). The remaining tumors were labeled as non-*BRCA1*(-)/*NF-κB*(+) (“*NF-κB* off”). Of the 42 genes identified and refined using ElasticNet computational analysis, 39 were upregulated in the “*NF-κB* on” subgroup. To investigate the clinical significance of the “*NF-κB* on” and “*NF-κB* off” subgroups, ElasticNet derived 42 gene signatures which were applied to 4 additional TNBC datasets. This application showed the “*NF-κB* on” subgroup has significantly better relapse free survival in the publicly available GSE58812 (HR = 0.2886, 0.1179–0.7065 95% CI, *p* = 0.0065), GSE21653 (HR = 0.1956, 0.04632–0.8263 95% CI, *p* = 0.0264), and GSE2034 (HR = 0.4412, 0.1929–1.009 95% CI, *p* = 0.0525) datasets. The “*NF-κB* on” subgroup expressed higher levels of genes associated with high ROS levels, which is consistent with the *in-vitro* studies discussed above [73].

Denosumab, a monoclonal antibody inhibitor for RANKL and NF-κB signaling, has been FDA approved for the treatment of breast cancer with bone metastases [79] (Table 1). In a stage 3 study named D-CARE, denosumab was given as an adjuvant to chemotherapy for stage II or II breast cancer over a span of 5 years. Although preclinical studies did show that bone metastases were decreased in patients with denosumab, this study did not show any evidence for improvement in outcomes in the addition of denosumab [80]. Additionally, another study also observed the effects of denosumab in hormone receptor positive breast cancer patients. Disease-free survival for 3,425 patients was determined for both adjuvant denosumab and placebo groups. At 5 years, the disease-free survival rate was significantly higher with denosumab at 89.2% (95% CI 87.6–90.8) and placebo at 87·3% (85.7–89.0) (HR 0.82, 95% CI 0.69–0.98, Cox *p* = 0.0260). This study illustrates the beneficial effect of denosumab in combination with aromatase inhibitor therapy [81].

**TABLE 1:** Summary of the discussed FDA-approved cancer drugs that inhibit NF-κB signaling and their mechanism of action.

Drug name	Drug class	Uses	Mechanism of action
Denosumab	Monoclonal antibody	Breast cancer with bone metastases, multiple myeloma	RANKL inhibition
Vorinostat, Romidepsin	Histone deacetylase inhibitor	Cutaneous T-cell lymphoma	Downregulation of proteasome subunit
Bortezomib, Carfilzomib	Proteasome inhibitor	Multiple myeloma, lung cancer	Inhibition of IκB degradation

Other mechanisms of NF-κB inhibition include acetylation which has been targeted in the treatment of cutaneous T-cell lymphoma (CTCL). Vorinostat and Romidepsin are acetylation inhibitors that downregulate activity of NF-κB in CTCL and are FDA-approved agents for the treatment of CTCL [18, 82, 83]. Other FDA approved agents that modulate NF-κB for multiple myeloma include proteasome inhibitors, Bortezomib and Carfilzomib. Proteasome inhibitors prevent the degradation of IκBα which then prevents activation of NF-κB, inhibiting tumorigenic activity [84]. Preclinical studies also led to further investigation of bortezomib in breast cancer patients. In a phase II clinic study of twelve patients with metastatic breast cancer treated with bortezomib as a single agent, no objective clinical responses were noted. There was, however, a significant reduction in IL-6 levels from an average level of 44.1 ± 12.7 units to 14.9 ± 5.5 units (*p* = 0.04). Decreased levels of IL-6 have corresponded with apoptosis, longer survival rates and reduced inflammation. This mechanism of NF-κB inhibition can be harnessed in the treatment of breast cancer alongside antitumor agents [85]. Further investigation of NF-κB could provide evidence of interaction with immune system activity of T-cells.

## NF-κB Immunotherapy

As discussed previously, NF-κB can have potent effects in signaling pathways involving the immune system, including GM-CSF. In previous studies, GM-CSF has been shown to mediate immune cytokines and upregulate the immune response against tumors with dendritic cell activation and T-lymphocyte activation [6]. One example of GM-CSF employment in cancer therapeutics includes Vigil, an autologous tumor cell vaccine transfected *ex vivo* with GM-CSF DNA and bifunctional short hairpin RNA against furin. Furin knockdown is known to suppress TGFβ1 and TGFβ2 [86]. Previous literature has shown the safety and efficacy of Vigil in various solid tumors [87–92]. In a phase IIb trial stage III/IV high grade serous, endometroid, or clear cell ovarian cancer, patients were observed to have an improved recurrence free survival compared to placebo, although not significant (11.5 vs. 8.4 months HR 0.69 CI 0.44–1.07 *p* = 0.078). More interestingly, Vigil is the first immunotherapy to show efficacy in the *BRCA* wild type population as the preplanned subgroup analysis results were statistically significant when compared to placebo (HR 0.51 CI 0.30–0.88 *p* = 0.02) [93]. Normal BRCA1 expression would also promote NF-κB expression at baseline [71, 73]. Vigil could block the immunosuppressive effects of TGFβ transcription and further inhibit proliferative NF-κB signaling and selectively increasing GM-CSF expression to enhance the anti-tumor effects [57, 94].

Within recent years, it has been discovered that NF-κB plays a role in the expression of genes known to assist in the evasion of immune responses and promotion of tumor survival, including through *PD-L1* transcription and post-translational expression [95]. Inflammatory cytokines, such as IFN- γ, IL-17, and TNF-α, are able to activate NF-κB-dependent pathways leading to PD-L1 upregulation, which in turn blocks immune checkpoint [96–99]. These effects on PD-L1 expression have been described in a variety of cancers and through a wide variety of mechanisms. For example, one study found that LPS, a pathogen-associated molecular pattern (PAMP), increases NF-κB activation, which results in PD-L1 upregulation in gastric cancer cells. This same study indicated that NF-κB regulates *PD-L1* gene transcription through p65-binding to the *PD-L1* promoter to increase gene expression [98]. Another study found that in melanoma cells, IFN- γ induces translocation of NF-κB, inducing *PD-L1* promoter activity and expression [100]. In ovarian cancer cells, the proto-oncogene *Bcl3* enables NF-κB p65 acetylation and p300-dependent recruitment to the *PD-L1* promoter resulting in increased *PD-L1* gene transcription [101]. Finally, Mucin1 (MUC1), an oncoprotein expressed in carcinomas of epithelial origin [102], activates pathways that lead to nuclear translocation of NF-κB and directly bind to NF-κB to drive transcription of *PD-L1* in TNBC [103]. Other cancers, including prostate cancer, colon cancer, and non-small cell lung carcinoma (NSCLC), have also demonstrated how NF-κB -induced expression of PD-L1 may be an attractive target in cancer therapeutics [96, 104].

Conversely, the inhibition of NF-κB activity is associated with suppression of *PD-L1* gene expression. inhibition of NF-κB in nasopharyngeal carcinoma cells was found to decrease expression of PD-L1 in a dose-dependent manner [105]. Additionally, CDK 4/6 phosphorylation of RB allows RB to interact with NF-κB p65, inhibiting NF-κB activity and suppressing PD-L1 gene expression. This RB-mediated suppression of PD-L1 gene expression is inhibited following radiation, resulting in the upregulation of PD-L1 transcription and expression [106, 107]. The clear relationship between NF-κB and PD-L1 expression in cancers has prompted the idea that perhaps siRNA-mediated *NF-κB* silencing or pharmacological inhibitors of NF-κB could be used as potential therapeutics [108]. Furthermore, there may be a targetable interaction between EGFR and NF-κB signaling that may result in decreased PD-L1 expression, though previous studies have shown conflicting data. EGFR appears to be involved in the regulation of post-translational PD-L1 expression, and EGFR stimulation was shown to stabilize PD-L1 in breast cancer cells *via* glycosylation. Without this glycosylation, PD-L1 is instead phosphorylated by glycogen synthase kinase 3-β, leading to its ubiquitination and degradation [109]. In breast cancer, TNF-α induces activation of p65, which binds to the *COPS5* gene promoter leading to enhanced transcription of *CSN5* and de-ubiquitination activity. CSN5 binds to PD-L1, causing the removal of PD-L1-bound ubiquitin which prevents degradation and promotes stability [110]. Similar patterns have been noted in nasopharyngeal carcinoma and colorectal cancer [111, 112]. CDK phosphorylation and EGFR signaling manipulation may provide two potential methods of inhibiting NF-κB and PD-L1 expression.

Recent advances in immunotherapy have shown benefit in breast and ovarian cancer patients. NF-κB involvement in the tumor microenvironment has been thoroughly studied in preclinical models, and clinical studies are ongoing to explore NF-κB in immunotherapy. A recent phase 1B/2 trial in non-small cell lung cancer (NSCLC) studied the effects of nivolumab, an anti-PD-1 inhibitor, combined with denosumab, a monoclonal antibody inhibiting receptor activator of NF-κB ligand (RANKL).

Although results are pending, the study could provide sufficient reasoning for involvement of NF-κB with immunotherapy [113]. In addition, an observational study reported outcomes after the use of immunotherapy with ipilimumab, pembrolizumab or nivolumab, combined with denosumab in melanoma and NSCLC patients. Longer exposure time to combination therapy showed improved survival in NSCLC (*p* < 0.001) [114]. Prospective studies should be conducted based on these preliminary results showing the capability of NF-κB inhibition to improve patient responses to immunotherapy. Another upcoming study will examine denosumab in ovarian cancer patients with *BRCA1* mutation, using immunohistochemistry of patient samples measuring Ki67 as a proliferation index to compare proliferation in denosumab vs. control groups [115]. Moreover, these results could be applied in breast and ovarian cancer and further research in clinical trials could be warranted.

## Discussion

NF-κB signaling plays an important role in cancer initiation, progression, and metastasis. In breast cancer, RANKL activates NF-κB, inducing cellular proliferation by targeting cyclin D1 gene [17]. Survival is also mediated by NF-κB through increased inhibitors of apoptosis (IAPs) and Bcl-xL. As for ovarian cancer, NF-κB activation of the oncogene PI3K-110α which can then increase p65RelA that is associated with increased chemoresistance and poor outcomes in patients with epithelial ovarian cancer [52, 53]. Many other immune signaling pathways, including EGFR, have also been shown to be mediated through NF-κB as discussed in this paper. Furthermore, breast and ovarian cancer can be found at higher rates in patients with BRCA1 mutations that are also affected by NF-κB signaling [71, 72]. This reasoning warrants investigation into immunotherapy, such as Vigil and PD-L1 inhibition, that can inhibit the effects of NF-κB found in chemoresistance associated with breast and ovarian cancer. NF-κB mediated inhibition should continue to be studied with an emphasis on recent immunotherapy which would lead to improvements in cancer therapeutics.
